# The Effect of an Innovative Financing and Payment Model for Tuberculosis Patients on Health Service Utilization in China: Evidence from Hubei Province of China

**DOI:** 10.3390/ijerph16142494

**Published:** 2019-07-12

**Authors:** Junnan Jiang, Henry Lucas, Qian Long, Yanjiao Xin, Li Xiang, Shenglan Tang

**Affiliations:** 1 School of Medicine and Health Management, Huazhong University of Science and Technology, Wuhan 430030, China; 2 Institute of Development Studies, University of Sussex, Brighton BN1 9RE, UK; 3 Global Health Research Center, Duke Kunshan University, Kunshan 215316, China; 4 Duke Global Health Institute, Duke University, Durham, NC 27710, USA

**Keywords:** China–Gates TB Project, Propensity score, Utilization, Health care services

## Abstract

*Background*: Tuberculosis (TB) remains a major social and public health problem in China. The “China–Gates TB Project” started in 2012, and one of its objectives was to reduce the financial burden on TB patients and to improve access to quality TB care. The aims of this study were to determine if the project had positive impacts on improving health service utilization. *Methods*: The ‘China–Gates TB Project’ was launched in Yichang City (YC), Hubei Province in April 2014 and ended in March 2015, lasting for one year. A series of questionnaire surveys of 540 patients were conducted in three counties of YC at baseline and final evaluations. Inpatient and outpatient service utilization were assessed before and after the program, with descriptive statistics. Propensity score matching was used to evaluate the impact of the China–Gates TB Project on health service utilization by minimizing the differences in the other characteristics of baseline and final stage groups. Focus group discussions (FGDs) were held to further enrich the results. *Results*: A total of 530 patients were included in this study. Inpatient rates significantly increased from 33.5% to 75.9% overall (*p* < 0.001), with the largest increase occurring for low income patients. Outpatient visits increased from 4.6 to 5.6 (*p* < 0.001), and this increase was also greatest for the poorest patients. Compared with those who lived in developed counties, the overall increase in outpatient visits for illness in the remote Wufeng county was higher. *Conclusions*: The China–Gates TB Project has effectively improved health service utilization in YC, and poor patients benefited more from it. TB patients in remote underdeveloped counties are more likely to increase the use of outpatient services rather than inpatient services. There is a need to tilt policy towards the poor, and various measures need to be in place in order to ensure health services utilization in undeveloped areas.

## 1. Introduction

China is one of the 30 countries with the highest tuberculosis (TB) burden. According to the National TB Epidemiology Survey conducted in 2017, there were approximately 900,000 new TB cases in China. This amount accounted for 9% of the total number of new tuberculosis patients worldwide and was the second largest TB epidemic in the world [1,2]. Due to the serious situation of TB in China, many interventions have been implemented in recent years, including the nationwide scale-up of the WHO-recommended the directly observed therapy, short-course (DOTS) strategy and a comprehensive program for multi-drug resistant tuberculosis (MDR-TB). Such initiatives have led to an impressive decline in pulmonary TB [3].

However, several factors add to the financial barriers for poor patients to access TB treatment in China. One of the main factors is poverty [4]. Due to the economic costs involved, the poor are more likely to develop tuberculosis and face greater financial barriers to quality treatment. A national TB survey in China in 2010 showed that 10% of tuberculosis patients received intermittent treatment, and another 22% discontinued before completing the treatment process [5]. Around 68% patients finished their regular TB treatment in China. Compared with 76% in Timor-Leste [6] and 83.3% in Indian [7], it was much lower than other low- and middle-income countries (LMICs). Economic difficulties were the reason for their abandonment of treatment, which was reported by 15% of patients who discontinued treatment. Research has also mentioned that poverty can lead to interruptions in TB treatment, especially in the first two months of starting treatment [8,9]. In addition, geographic distance significantly influences the seeking of active TB treatment [10]. Patients living in remote rural areas face more difficulties in early service utilization. They must consider the costs of transportation and accommodation involved in seeking outpatient or inpatient services [11]. Another factor, public health financing programs, was proven to be important for TB treatment, especially in LMICs where the resource is limited [12]. Effective public health financing could alleviate the financial burden of TB patients and avert most cases of poverty [13]. 

In the context of universal health coverage, the China National Health and Family Planning Commission (NHFPC)–The Bill & Melinda Gates Foundation TB Phase II program (the ‘China–Gates TB Project’) was implemented between 2012 and 2015 in three prefectures in three cities in China. The new TB financing and payment model included increasing health insurance reimbursement rates for hospitalization and outpatient TB services, changing the provider payment method to a case-based payment from the current fee-for-service, and providing transportation and subsistence financial incentives to TB patients who adhered to treatment. The components were documented in a previous study [14].

TB is a communicable disease that is often associated with poverty and hardship, and disparities in terms of both disease prevalence rate and access to diagnosis and treatment are well established and widespread [15,16]. Many previous studies have examined equity issues related to geographic locations, gender, and the socio-economic status of TB patients [17,18,19,20]. Moreover, rich international literature has suggested that public subsidies for health programs frequently benefit richer people more than poorer people [21,22,23]. Whether a health insurance-based approach can effectively target the poor is therefore of great concern. In this study, we provide an evaluation of the China–Gates TB Project on TB health utilization in a central area of China. We aimed to determine (1) whether the China–Gates TB Project increased outpatient visits and inpatient hospitalization utilization rates and (2) whether the China–Gates TB Project increased the use of TB health services similarly across income and county subgroups. Our findings quantitatively and qualitatively assess tuberculosis financing and payment model performance relative to the health improvements and may contribute to improving and adjusting the China–Gates TB Project policy, thereby further relieving the inequity in healthcare.

## 2. Materials and Methods

### 2.1. Setting

The China–Gates TB program Phase II was implemented in three prefectures from eastern (Zhenjiang), central (Yichang) and western (Hanzhong) China. According to the study of Meng Q. et al. [24], central regions and rural areas may be more sensitive to health policies. With the health reform implemented on the China mainland, the largest increases of outpatient care and hospital admissions over an 8-year period occurred in the central region and rural areas by 2011 [25]. Thus, this paper focuses on TB services utilization in the rural areas of central China, such as Yichang. 

Yichang City is situated in the southwest of Hubei province and had a GDP per capita of 93,394 Chinese Yuan (CNY) (US$ 14,991) in 2017, ranking 2nd among the 17 cities of Hubei. As of the end of 2017, the resident population of Yichang City was 4,135,600. There are 13 counties/districts in Yichang city. Table A1 (Appendix A) shows the detail of the China–Gates TB program Phase II in Yichang city. For this study, the counties/districts of Yichang City were stratified into high, middle and low GDP per capita, and one county was randomly selected from each stratum. 

**Figure 1:**
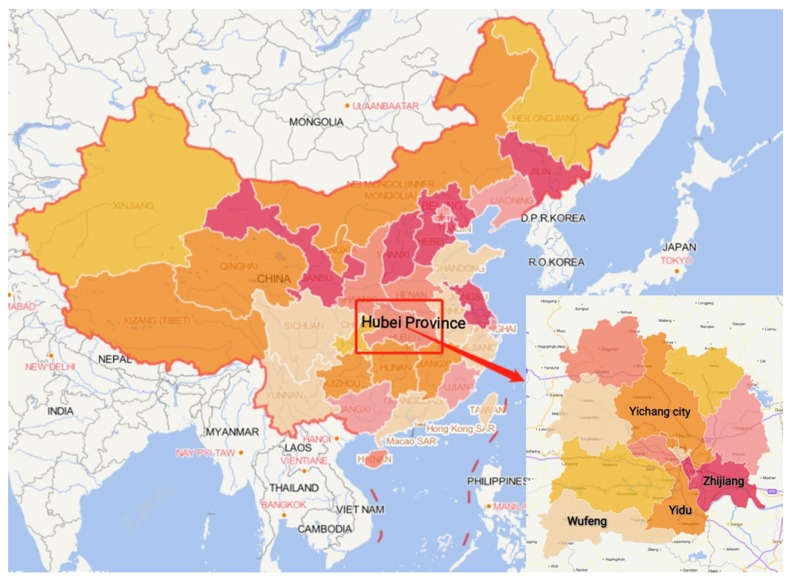
Location of sample counties in Yichang city. Data source: Map World, https://zhfw.tianditu.gov.cn/.

Wufeng (WF), Zhijiang (ZJ), and Yidu (YD) were chosen as the sample counties (Figure 1). As one of the most developed counties in Yichang City, Yidu (YD) had a GDP per capita of almost 130,000 CNY (US$ 20,867) in 2015. In 2012, the TB prevalence of YD was 105.8/million, and the local tuberculosis designated medical institution had 3 tuberculosis specialists and 9 specialist nurses. Zhijiang is situated on the left bank of the Yangtze River, with a GDP per capita approximately 80,000 CNY (US$ 12,841). Before the program, the TB prevalence of ZJ was 79.8/million. There were 3 TB doctors and 3 nurses in local department of infectious diseases in 2012. Wufeng is a relatively underdeveloped area, with a GDP per capita of some 30,000 CNY (US$ 4815); it is an ethnic minority, mountainous county with an average elevation of 1100 m. The TB prevalence of WF was 84.3/million in 2012, and local TB hospitals had 2 specialists and 4 specialist nurses.

### 2.2. Data Collection

#### 2.2.1. Quantitative Data Collection

The ‘China–Gates TB Project’ was launched in April 2014 and ended in March 2015, lasting for one year. In order to measure the effectiveness of the project, we conducted sample surveys of patients before and after the intervention. The minimum required sample size per city was calculated to be 264 cases by using selected key indicators (the financial burden of TB care and treatment adherence). Thus, we planned to obtain 540 cases before and after the intervention (270 in baseline, 270 in terminal stage). Probability proportional to size (PPS) sampling [25] was adopted in selecting 90 TB patients from each county. In the baseline stage, patients who had had completed the normal treatment or stop treatment during 2012 were selected from a TB case registration list. In the terminal stage, we sampled by the same size and method according to the baseline survey. Patients were required to have registered between April 2014 and March 2015 and completed a treat course of six months or more. In both stages, the inclusion criteria required that patients were drug-sensitive and had been diagnosed at least 6 months prior (8 months for relapse patients). Patients with communication barriers, who worked in another city within the study period (those patients cannot be involved in this intervention and may mix research results and overestimation of policy effects), and who had mental illnesses or multidrug-resistant TB were excluded from this study. The baseline survey was conducted in August 2013, and the final assessment survey was organized in July 2015.

A standardized questionnaire was used for the survey. It was constructed with reference to the National Health Services Survey Questionnaire and the China Health and Retirement Longitudinal Study (CHALS) Questionnaire [26]. It included questions on the demographic data of patients and their households, as well as the utilization of outpatient and inpatient services. Outpatient visits were defined as the total number of outpatient visits by TB patients to a health facility during the past year, and inpatient care was defined as any hospitalization for at least one day during the last year. After being trained in interview skills and the contents of the questionnaire, students from local universities conducted patient survey interviews. All completed questionnaires were independently examined to identify and correct errors.

##### Ethics Approval and Consent to Participate

Our questionnaire had relevant patient informed consent and choice in the heading place, and we obtained written consent from the patient. Ethical approval was obtained from the Institutional Ethics Committee, Chinese Center for Disease Control and Prevention, China (No.201626).

#### 2.2.2. Qualitative Data Collection

In this study, we conducted several face-to-face focus group discussions (FGD) to enrich the results. The FGDs consisted of two parts: (1) A group discussion with local hospital staff including 1–2 outpatient physicians, 1–2 nurses from TB units, and 2–3 inpatient physicians—issues posed included the provision of TB services, TB patients’ management, and project implementation problems; (2) a group discussion with 5–6 TB patients. In order to better understand the use of inpatient services, the inclusion criteria were patients with previous TB hospitalization treatment who could clearly communicate their thoughts. The reasons for doing the research were introduced to participants firstly. Questions regarding outpatient and inpatient service utilization, the effects of the project, and difficulties in service utilization were asked. A total of 11 FGDs were conducted by university professors with rich qualitative research experience and experts invited by China local Center for Disease Control (CDC) in a local TB hospital. We recorded interviews with the permission of the participants. Detailed field notes were made during the focus groups. Consolidated criteria for reporting qualitative studies (COREQ) were checked (Appendix A Table A2).

### 2.3. Analysis

To estimate the impact of the China–Gates TB program Phase II, we applied the method of propensity score matching (PSM). PSM was first introduced by Rosenbaum and Rubin [27] and has been proven to being useful in the evaluating policy impact [28]. The purpose of matching is to find those individuals in a large group of non-participants that are similar to the participants in observed pre-processing features, minimizing the selection bias associated with non-experimental data. If there is no difference in the relevant unobservable factors between the two groups, the difference in results between the two groups can be attributed to the policy [29]. This matches the group of treated and untreated subjects with similar propensity score values. In this study, propensity scores were assigned for each TB patient based on logistic regression using patient characteristics, including gender, age, marriage, education, and insurance type. The Kernel matching (KM) [30,31] method was adopted to estimate the average treatment effect (ATT). This method has been used widely in related studies reported from Colombia [32] and China [33]. The t-test for equality of the means and the standardized bias were calculated as a balancing test [34]. The results show that all indicators were less than 10% after matching. At the same time, compared with the results before matching, the standardization variation of most variables was greatly reduced, indicating a reasonable comparability between treated and untreated groups [35]. The Kernel density estimate of the probability distribution of propensity scores before and after matching was also examined. Standard errors through bootstrapping with 500 replications were calculated. To further compare the impacts of the project among income and counties, the ATT was calculated in income subgroups and county subgroups separately. Medical expenses and income before and after the intervention were discounted according to the local average income. Data transformation and statistical analyses were performed using Stata 12.0 (StataCorp LP., College Station, TX, USA). 

Because the main measure of the innovative financing and payment model was to increase the outpatient and inpatient health insurance benefits of TB patients (Appendix A, Table A1), we added the local health insurance expenditures on TB patients before and after the intervention, as the costs and calculated the cost-effectiveness by this formula:

Cost−effectiveness=Pre−post change Health insurance expenditures increment*1000

Qualitative interview recordings were transcribed into textual information, and the respondent’s personal information was deleted to ensure anonymity. For some textual ambiguity, the research team consulted and repeated checks and verifications to ensure that the meaning expressed by the respondent was understood as accurately as possible. We used a framework approach to analyze qualitative data [36]. The analysis framework was developed based on the topic guide and emerging themes from the transcripts and was refined during the coding process. All qualitative data were coded, sorted, and classified in terms of the analysis framework. Under the four major themes of the TB prevention and control system—the implementation of the financing and payment systems, as well as the new policy’s impact and supervision (all of which were determined in advance)—we sorted out the views of different stakeholders. We used the original Chinese texts for analysis and translated the quotations into English. The analysis was conducted using NVIVO 9.0 (QSR International Pty Ltd., Melbourne, Australia).

### 2.4. Quality Assurance

Prior to the investigation, there was a pre-test which tested all of the data collection instruments, tools and procedures in the pilot area, and then analyzed the results and adjusted the tools. All collected data were also logically checked to determine gaps, inaccuracies, and apparent inconsistencies.

## 3. Results

### 3.1. Demographic, Socio-Economic Characteristics of TB Patients

Table 1 shows the social-economic characteristics of the TB patients. In total, 530 patients were included in this study, 260 in the baseline stage and 270 in the final stage. The mean ages were 53.5 and 56.9, respectively. There were 71 women in both study populations, less than half the number of men. Most patients were married (75.8% and 77.4%, respectively). Approximately 60% were employed, but their degree of education was limited, with approximately 45% and 50%, respectively, having only primary school or lower. Most baseline TB patients were insured with the New Cooperative Medical Scheme (NCMS) (93.1%), and the percentage with Urban Employee Basic Medical Insurance (UEBMI) and Urban Resident Basic Medical Insurance (URBMI) increased in the final evaluation. The average household income was 37,865 CNY (US$ 6078) in 2013 and 29,434 CNY (US$ 4725) in 2015 (exchange rate 623 CNY = US$100 at the end of 2015). 

After the intervention, the mean of outpatient visits for total TB patients from the three counties increased from 4.6 to 5.6 (Table 1), which was statistically significant at the 1% level. Approximately 33.5% of 260 individuals reported a hospitalization during the one-year recall period at baseline, and this proportion significantly increased to 75.9% after the project (*p* < 0.001).

“Before the ‘China–Gates TB Project,’ the outpatient cost was non-reimbursable, and I underwent TB treatment for four months and then stopped treatment because of high cost. But this time, we just paid 780 CNY in the first month, and every time you take drugs, you don’t have to pay until to six months later. I had finished this treatment.”

(TB patients, FGDs)

Table 2 denotes the pre-post health utilizations of TB patients of each county. We found that the TB patients of WF had the greatest growth of outpatient visits through the project. Before the program, the average outpatient visit of WF was 3.01, and it rapidly increased to 4.59 after the intervention. Each additional 1000 CNY invested by local medical insurance on outpatient services can increase the number of outpatient visits by 2.435 times. For the inpatient rate, TB patients in YD had the highest increase, from 0.12 to 0.80. The cost-effectiveness of ZJ, YD and WF were 0.374, 0.440, and 0.094, respectively.

**Figure 2:**
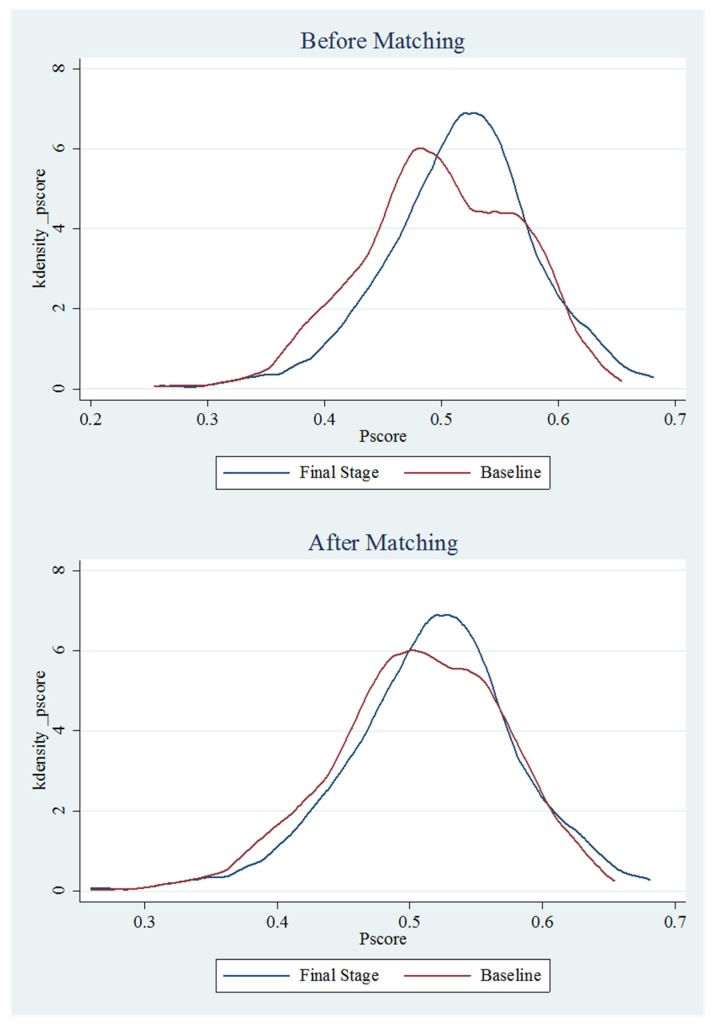
Overlay of Kernel density distributions of baseline and final stage propensity scores before and after propensity score matching.

Figure 2 shows that most observations are within a common support area to perform PSM, and the partial area was further increased after matching.

### 3.2. The Decision to Use Health Services

Table 3 reports the results of the propensity score matching to assess the impact of new projects on the use of TB medical services in the total sample from the three counties. Compared with the baseline group, the overall outpatient visit rate of the final stage group increased by 106%. The hospitalization rate in the final stage group increased by 43% compared with the baseline group (*p* < 0.001).

Table 4 and Table 5 show the propensity score matching results on the average treatment effect (ATT) in TB health care utilization by income and county subgroups. The growth of outpatient visits was more pronounced in low-income populations. Outpatient visits were found to have increased by approximately 121% among the poorest quartile. Similarly, the proportion of hospitalization also showed the same trend. At the county level, outpatient visits increased the most in WF (ATT = 1.491, *p* < 0.001), while ZJ had the smallest increase (ATT = 0.188, *p* = 0.578). Inpatient care increased by approximately 67.9% and 22.4% among the final stage compared to the baseline group in YD and WF, respectively.

“Tuberculosis patients are mostly farmers, and their income is relatively low. In the past, some patients had poor compliance with the treatment. Because of economic problems or other reasons, they would not come or be interrupted without treatment. After the project is launched, standard treatment can be guaranteed.”

(Physicians in hospitals, FGDs)

“I had received medical financial assistance because I belonged to local eligible low-income household. They gave me 500 CNY last year. The reimbursement rate of hospitalization was approximately 80%, and I got nutrition subsidy, approximately 60 CNY per month. Anyway, this new policy is very good.”

(TB patients, FGDs)

“Outpatient clinics only need to pay for medicines, but other hospitalization expenses, such as hospitalization living expenses, are relatively high. If you live in a month, you would spend more than 2,000 CNY. The mountain traffic is not very convenient, round trip to 30 CNY or more. Or the outpatient service is more convenient.”

(TB patients in WF, FGDs)

“I preferred to be hospitalized, because I can claim higher reimbursement rates. Outpatient reimbursement rates was low. And the doctors at our county have a particularly bad attitude and are very impatient. I don’t really want to go to them to see the clinic.”

(TB patients in ZJ, FGDs)

## 4. Discussion

Overall, the China–Gates TB program Phase II was found to improve TB health utilization, no matter the outpatient visit or inpatient admission rates. The program effects were greater for the low income groups compared to the richest group. The effect of the intervention was significantly different between counties. The number of outpatient visits increased the most in the remote county (WF).

Case-based payments contributed to the increased TB health utilization after the project. New payment methods effectively decreased the out-of-pocket medical payments of TB patients. For outpatient visits, rural TB patients were basically treated at their own expense with a low outpatient reimbursement rate before the project was launched. After the project, patients only paid 20% of the total medical expenditure for outpatient visits, with a 780 CNY (US$ 125) cap. Qualitative interviews also proved this result. Furthermore, “fee-for-service” payment has been proven to be another major factor affecting the use of TB health services in China [37]. Physicians in different health facilities would prescribe repeated examinations and treatments with income incentives, causing a delay of TB diagnosis, a high cost, and poor effects of the treatment. The shift from fee-for-service to case-based payment can effectively reduce the negative impression of patients and promote the use of outpatient and inpatient services.

Compared with the rich, the hospitalization and outpatient services of the poor increased more. With rich interview material from FGDs, the pro-poor effects can be explained by two reasons: On the one hand, transport and subsistence allowances provided strong incentives to poor patients, many of who live in remote rural areas and have higher transportation costs, to seek care. Financial or material incentives such as food, transportation subsidies, and/or money were considered to be effective at reducing the direct and opportunity costs of treatment [38,39]. Travel subsidies to get to a clinic for TB patients were used in other studies in China [40,41] but did not reach the poor tuberculosis patients because they provided an inadequate amount (only US$ 1 for the first visit). In this project, about US$ 10 were given to TB patients per month. Higher financial incentives could perform better. On the other hand, medical financial assistance was provided by the Department of Civil Affairs for eligible low-income households, further reducing their financial burden. 

Why did the higher incomes group not have the most growth? For the wealthier patients, there is less potential health services demand. The price elasticity of health service demands is small. Even if the reimbursement rate was increased, they would not significantly increase their utilization of health services. This result is inconsistent with many other studies. Rao and Peters [42] noted that public health interventions sometimes benefit the wealthy, given that the better-off are more likely to use health services when they are ill. Victora et al. [43] found that maternal and child health interventions initially increased but then reduced inequality. The interventions were first accessed by richer households but were then taken up by the poor. The reasons for the difference may be that there are special subsidies that tilt the policies to benefit the poor in this project.

The difference in effect between counties is obvious. As an underdeveloped mountainous county, patients in WF preferred to increase outpatient services rather than inpatient ones. Qualitative results from the study indicate several possible reasons. TB patients generally go to the outpatient clinics in the county and go to the Yichang municipal medical institutions for hospitalization. The cost of outpatient visits is lower, and due to their living in an undeveloped area, tuberculosis patients in WF are more sensitive to outpatient services. They also want to make more use of inpatient services, but the cost of hospitalization is high. In addition, hospital accommodation, transportation, accommodation and other expenses are also economic barriers. WF is the farthest county away from Yichang city. The mountainous terrain has caused local traffic inconveniences. If patients go to the city to seek inpatient services, the cost of transportation and accommodation will be much higher than for other counties. In the rural mountainous areas of China, limited transportation, poverty and poor primary health services may make medical services for diagnosis and treatment of tuberculosis worse [44]. In terms of cost-effectiveness, the differences between counties are still obvious. WF had the best cost-effectiveness in outpatient intervention, while YD had the best in inpatient services utilization. This finding suggests that we should consider regional differences when implementing projects and give more transportation and living subsidies to counties with inconvenient transportation. The FGDs also showed physicians’ attitude was another reason for outpatient visits. The increased outpatient visits and inpatient admission rate suggest that TB health services utilization improved. However, does it truly indicate a ‘good’ improvement? Currently, TB treatment guidelines by the WHO and China CDC recommend that rifampicin-sensitive newly diagnosed TB patients should receive six months of outpatient treatment, and relapsed TB patients should receive eight months. The increase in the overall average number of outpatient visits from 4.6 to 5.6 implies that more TB patients received outpatient treatment in line with the standard (six-to-eight visits) recommended by the WHO guideline and the standard of outpatient diagnosis and treatment of tuberculosis in China [45], which can be seen as a beneficial change. However, the sharp rise in the inpatient rate, from 33.5% to 75.9%, is contrary to the recommended treatment guidelines [46,47], which indicate that only patients with serious complications or severe adverse reactions require hospitalization. Compared with other studies, the observed inpatient rate appears excessive. For example, the hospitalization rate of TB patients was 54% in Montreal, Canada [48] and 66% in Spain [49]. High impatient rates increase the financial burden on patients and may overload treatment facilities [50]. Thus, this apparent improvement in inpatient services utilization cannot be seen as uniformly beneficial. 

This study has several limitations. First, there was very limited quantitative information on patient case-mix and service details (such as prescriptions and procedures). Therefore, the appropriateness and quality of services cannot be assessed objectively. Second, the study was not a randomized controlled study. We evaluated the impact of the new project with pooled cross-section data before and after intervention. However, various biases may occur in implementation. China is implementing health system reforms, and there were multiple concurrent policy interventions that may be synergistic or antagonistic. These confounding factors may lead to a biased estimate of the role of the China–Gates TB Project on the outcomes. Thirdly, the results of this study can provide a reference for areas with similar economic development levels in central China. However, considering that the study included three counties within one city in China, there are limits to extending the results of this sample to all regions.

## 5. Conclusions

This study provides additional evidence to policy makers that the China–Gates TB Project does help to improve TB patients’ access to inpatient and outpatient services in YC. Patients in households classified as the lowest income group benefited more from the program compared to the high income group, indicating an improved equity in TB service access. TB patients in remote, underdeveloped counties are more likely to increase their use of outpatient services rather than inpatient services. This study suggested that the implementation of the China–Gates TB Project is necessary, and the Chinese government urgently needs to place various measures which lean toward the poor and ensure health services utilization in undeveloped areas.

## Figures and Tables

**Table 1:** Descriptive statistics of variables in the baseline and final stage.

Variables	Baseline (*n* = 260)	Final Evaluation (*n* = 270)	*p*-Value
**Independent variables**	*n* (%)	*n* (%)	
**Gender**			
Male	189 (72.7)	199 (73.7)	0.793
Female	71 (27.3)	71 (26.3)	
**Age (mean (Std))**	53.5 (15.0)	56.9 (13.9)	0.006
**Marriage**			
Married	197 (75.8)	209 (77.4)	0.656
Others	63 (24.2)	61 (22.6)	
**Patient category**			
New patient	223 (85.8)	231 (85.6)	0.944
Recurrent patient	37 (14.2)	39 (14.4)	
**Education level**			
None	30 (11.5)	37 (13.7)	0.655
Primary	87 (33.5)	99 (36.7)	
Secondary	107 (41.2)	101 (37.4)	
High school and above	36 (13.8)	33 (12.2)	
**Employment**			
Currently working (including farming)	164 (63.1)	153 (56.7)	0.142
Others	96 (36.9)	117 (43.3)	
**Insurance type**			
UEBMI	6 (2.3)	29 (10.8)	0.000
NCMS	242 (93.1)	217 (80.7)	
URBMI	8 (3.1)	20 (7.4)	
other insurance	4 (1.5)	3 (1.1)	
**Income level**			
1st (poorest)	54 (20.8)	75 (27.8)	0.128
2nd	63 (24.2)	69 (25.5)	
3rd	68 (26.2)	68 (25.2)	
4th Quartile	75 (28.8)	58 (21.5)	
**County**			
ZJ	89 (34.2)	92 (34.1)	0.224
YD	90 (34.6)	110 (40.7)	
WF	81 (31.2)	68 (25.2)	
**Outcome variables**			
Outpatient visits (mean (Std))	4.6 (2.0)	5.6 (1.4)	0.000 ^a^
Inpatient care	87 (33.5)	205 (75.9)	0.000

a: The t test.

**Table 2:** The pre-post health utilizations and cost-effectiveness of tuberculosis (TB) patients by counties.

County	Time	Outpatient Visits (a)	Outpatient Funds Expenditure /CNY (b)	Cost-Effective (= a/b*1000)	Inpatient Care (c)	Inpatient Funds Expenditure/CNY (d)	Cost-Effective (= c/d*1000)
ZJ	Pre-	5.81	353	0.054	0.47	3322	0.374
	Post-	5.96	3132		0.83	4284	
YD	Pre-	4.78	-	-	0.12	2619	0.440
	Post-	5.87	3022		0.80	4163	
WF	Pre-	3.01	524	2.435	0.42	3471	0.094
	Post-	4.59	1173		0.60	5386	

**Table 3:** Propensity score matching results on average treatment effect (ATT)in TB health care utilization.

Outcome Variables	Observed Coefficient (ATT)	Bootstrap Standard Error	*p* > |*z*|
Outpatient visits	1.059	0.150	0.000
Inpatient care	0.431	0.042	0.000

**Table 4:** Propensity score matching results on average treatment effect (ATT) in TB health care utilization by income quartiles.

Outcome Variables	Income Level	Observed Coefficient (ATT)	Bootstrap Standard Error	*p* > |*z*|
Outpatient visits	1st (poorest)	1.209	0.366	0.001
	2nd	1.400	0.471	0.003
	3rd	0.454	0.255	0.075
	4th Quartile	1.037	0.377	0.006
Inpatient care	1st (poorest)	0.416	0.103	0.000
	2nd	0.431	0.104	0.000
	3rd	0.324	0.103	0.002
	4th Quartile	0.377	0.104	0.000

**Table 5:** Propensity score matching results on average treatment effect (ATT) in TB health care utilization by counties.

Outcome Variables	County	Observed Coefficient (ATT)	Bootstrap Standard Error	*p* > |*z*|
Outpatient visits	ZJ	0.188	0.338	0.578
	YD	1.094	0.183	0.000
	WF	1.491	0.346	0.000
Inpatient care	ZJ	0.343	0.079	0.000
	YD	0.679	0.058	0.000
	WF	0.224	0.100	0.025

## Declarations

**Availability of data and materials.** The datasets used and/or analyzed during the current study are available from the corresponding author on reasonable request.

## Abbreviations

TB	Tuberculosis
**YC**	Yichang City
**FGDs**	Focus group discussions
DOTS	Directly Observed Therapy Short-course
**MDR-TB**	multi-drug resistant tuberculosis
**NHFPC**	National Health and Family Planning Commission
China–Gates TB Project	China National Health and Family Planning Commission (NHFPC)—the Bill & Melinda Gates Foundation TB Phase II program
**CNY**	Chinese Yuan
**WF**	Wufeng
**ZJ**	Zhijiang
**YD**	Yidu
**PSM**	propensity score matching
**KM**	Kernel matching
**ATT**	average treatment effect
**NCMS**	New Cooperative Medical Scheme
URBMI	Urban Resident Basic Medical Insurance
UEBMI:	Urban Employee Basic Medical Insurance
CHALS:	China Health and Retirement Longitudinal Study
WHO	World Health Organization
CDC	Center for Diseases Control

## Appendix A

**Table A1:** Tuberculosis financing and payment model of the China–Gates TB program Phase II.

Prospective	Contents
Outpatient services	Increased the reimbursement rate to 80%, patients need to pay up to 780 CNY for the entire treatment period.
Inpatient services	Case-based payments, increased the reimbursement rate to 80%, patients need to pay up to 900 CNY/1800 CNY (smear negative) for the entire treatment period.
Subsides	Supply nutrition and transportation assistance for eligible low-income households: 60 CNY/ month for nutrition and 10 CNY/month for transportation.
Others	Free sputum smear and chest X-ray examination for patients with suspected symptoms of tuberculosis, clinical pathways, and case management.

**Table A2:** Consolidated criteria for reporting qualitative studies (COREQ): 32-item checklist.

No Item	Guide Questions/Description
Domain 1: Research team and reflexivity	
Personal Characteristics	
1. Interviewer/facilitator	Methods, Qualitative data collection, paragraph 1
2. Credentials	N/A
3. Occupation	Methods, Qualitative data collection, paragraph 1
4. Gender	N/A
5. Experience and training	Methods, Qualitative data collection, paragraph 1
Relationship with participants	
6. Relationship established	N/A
7. Participant knowledge of the interviewer	Methods, Qualitative data collection, paragraph 1
8. Interviewer characteristics	N/A
Domain 2: Study design	
Theoretical framework	
9. Methodological orientation and Theory	Methods, Analysis, paragraph 4
10. Sampling	Methods, Qualitative data collection, paragraph 1
11. Method of approach	Methods, Qualitative data collection, paragraph 1
12. Sample size	Methods, Qualitative data collection, paragraph 1
13. Non-participation	N/A
Setting	
14. Setting of data collection	Methods, Qualitative data collection, paragraph 1
15. Presence of non-participants	N/A
16. Description of sample	Methods, Qualitative data collection, paragraph 1
Data collection	
17. Interview guide	Methods, Qualitative data collection, paragraph 1
18. Repeat interviews	N/A
19.Audio/visual recording	Methods, Qualitative data collection, paragraph 1
20. Field notes	Methods, Qualitative data collection, paragraph 1
21. Duration	Methods, Qualitative data collection, paragraph 1
22. Data saturation	Methods, Analysis, paragraph 4
23. Transcripts returned	N/A
Domain 3: Analysis and findings	
Data analysis	
24. Number of data coders	N/A
25. Description of the coding tree	N/A
26. Derivation of themes	Methods, Analysis, paragraph 4
27. Software	Methods, Analysis, paragraph 4
28. Participant checking	N/A
Reporting	
29. Quotations presented	Results, Demographic, socio-economic characteristics of TB patients, paragraph 3
30.Data and findings consistent	Discussion, paragraph 5
31.Clarity of major themes	Results, The decision to use health services, paragraph 3,4
32.Clarity of minor themes	N/A
